# Supplementary material for: Quality Standard for Rehabilitation of Young Deaf Children Receiving Cochlear Implants
Source: Medicina (Kaunas). 2023 Jul 24;59(7):1354. doi: 10.3390/medicina59071354 (PMC10386159; doi:10.3390/medicina59071354)
Supplement: Supplementary file 1 [file medicina-59-01354-s001.zip › Supplementary_Final list of publications scored on level of evidence A and B.pdf]

Overview publications of the systematic review on rehabilitation, auditory, speech and language development of young CI-children

A-score

1. Monshizadeh L, Vameghi R, Rahimi M, Sajedi F, Adegari;F., Hashemi SB. The effectiveness of a specifically-designed language intervention protocol on the cochlear implanted children's communication development. Int J Pediatr Otorhinolaryngol. 2019;109631.

2. Qiao X, Ren Q, X L, Li TL, Mariano R. Analysis of subjective perception and influencing factors of different inclusive education models among prelingually deaf children with a cochlear implant. J Int Med Res. 2020;48(6), 1-7.

B-score

1. Abdi S, Khalessi MH, Khorsandi M, Gholami B. Introducing music as a means of habilitation for children with cochlear implants. Int J Pediatr Otorhinolaryngol. 2001 Jun 7;59(2):105-13.

2.Archbold SM, Nikolopoulos TP, Lloyd-Richmond H. Long-term use of cochlear implant systems in paediatric recipients and factors contributing to non-use. Cochlear Implants Int. 2009 Mar;10(1):25-40.

3.Bakhshaee M, Ghasemi MM, Shakeri MT, Razmara N, Tayarani H, Tale MR. Speech development in children after cochlear implantation. Eur Arch Otorhinolaryngol. 2007 Nov;264(11):1263-1266.

4.Bavin EL, Sarant J, Hackworth NJ, Bennetts SK, Buzhardt J, Jia F, Button E, Busby P, Leigh G, Peterson C. Modelling the early expressive communicative trajectories of infants/toddlers with early cochlear implants. J Child Lang. 2020 Jul;47(4):796-816.

5.Bavin EL, Sarant J, Leigh G, Prendergast L, Busby P, Peterson C. Children with cochlear implants in infancy: predictors of early vocabulary. Int J Lang Commun Disord. 2018 Jul;53(4):788-798.

6.Castellanos, Irina; Kronenberger, William G; Beer, Jessica; Henning, Shirley C; Colson, Bethany G; Pisoni, David B. Preschool speech intelligibility and vocabulary skills predict long-term speech and language outcomes following cochlear implantation in early childhood. Cochlear Implants International: An Interdisciplinary Journal, Jul2014; 15(4): 200-210.

7.Chen Y, Wong LL, Zhu S, Xi X. Early speech perception in Mandarin-speaking children at one-year post cochlear implantation. Res Dev Disabil. 2016 Feb- Mar;49-50:1-12

8.Chen Y, Wong LL, Zhu S, Xi X. Vocabulary development in Mandarin-speaking children with cochlear implants and its relationship with speech perception abilities. Res Dev Disabil. 2017 Jan;60:243-255.

9.Ching TYC, Dillon H, Leigh G, Cupples L. Learning from the Longitudinal Outcomes of Children with Hearing Impairment (LOCHI) study: summary of 5-year findings and implications. Int J Audiol. 2018 May;57(sup2):S105-S111.

11. Compton, M.V., Tucker, D.A., Flynn, P.F. Preparation and perceptions of speech-language pathologists working with children with cochlear implants (2009) Communication Disorders Quarterly, 30 (3), pp. 142-154.

12.Connor CM, Hieber S, Arts HA, Zwolan TA. Speech, vocabulary, and the education of children using cochlear implants: oral or total communication? J Speech Lang Hear Res. 2000 Oct;43(5):1185-1204.

13.Davidson LS, Geers AE, Blamey PJ, Tobey EA, Brenner CA. Factors contributing to speech perception scores in long-term pediatric cochlear implant users. Ear Hear. 2011 Feb;32(1 Suppl): 19S-26S.

14.Deocampo, Joanne A; Smith, Gretchen NL; Kronenberger, William G; Pisoni, David B; Conway, Christopher M. The Role of Statistical Learning in Understanding and Treating Spoken Language Outcomes in Deaf Children With Cochlear Implants, Language, Speech & Hearing Services in Schools (Online), suppl. Special Issue: How Statistical Learning Relates to Speech-Language Pathology; Washington Vol. 49, Iss. 3S, (Aug 2018): 723-739.

15.De Raeve, L. (2010). A Longitudinal Study on Auditory Perception and Speech Intelligibility in Deaf Children Implanted under the Age of 18 Months, in Comparison to those Implanted at Later Ages. Otology & Neurotology, 31(8): 1261-1267.

16.De Raeve, L., Vermeulen, A., Snik, A. Verbal cognition in deaf children using cochlear implants: Effect of unilateral and bilateral stimulation (2015) Audiology and Neurotology, 20 (4), pp. 261-266.

16.Dettman SJ, Pinder D, Briggs RJ, Dowell RC, Leigh JR. Communication development in children who receive the cochlear implant younger than 12 months: risks versus benefits. Ear Hear. 2007 Apr;28(2 Suppl):11S-18S.

17.Dettman, Shani Joy; Dowell, Richard Charles; Choo, Dawn; Arnott, Wendy; Abrahams, Yetta; Davis, Aleisha; Dornan, Dimity; Leigh, Jaime; Constantinescu, Gabriella; Cowan, Robert; Briggs, Robert J. Long-term Communication Outcomes for Children Receiving Cochlear Implants Younger Than 12 Months: A Multicenter Study. Otology & Neurotology, Feb2016; 37(2): e82-e95.

18.Dettman, S., Wall, E., Constantinescu, G., & Dowell, R. (2013). Communication outcomes for groups of children using cochlear implants enrolled in auditory-verbal, aural-oral, and bilingual-bicultural early intervention programs. Otology & Neurotology, 34, 451-459.

19.Dillon CM, de Jong K, Pisoni DB. Phonological awareness, reading skills, and vocabulary knowledge in children who use cochlear implants. J Deaf Stud Deaf Educ. 2012 Spring;17(2):205-226.

20.Edwards, Lindsey; Anderson, Sara. The association between visual, nonverbal cognitive abilities and speech, phonological processing, vocabulary and reading outcomes in children with cochlear implants. Ear & Hearing, 2014 May-Jun; 35(3): 366-374.

21.Flipsen P Jr, Parker RG. Phonological patterns in the conversational speech of children with cochlear implants. J Commun Disord. 2008 Jul-Aug;41(4):337-357.

23.Geers A, Brenner C, Davidson L. Factors associated with development of speech perception skills in children implanted by age five. Ear Hear. 2003 Feb;24(1 Suppl):24S-35S.

25.Geers AE; Nicholas JG; Moog JS. Estimating the influence of cochlear implantation on language development in children. Audiological Medicine, Dec2007; 5(4): 262-273.

26.Geers A; Tobey E; Moog J; Brenner C. Long-term outcomes of cochlear implantation in the preschool years: from elementary grades to high school. International Journal of Audiology, Nov2008 Supplement 2; 47: S21-30.

27.Geers, Ann E; Davidson, Lisa S; Uchanski, Rosalie M; Nicholas, Johanna G. Interdependence of linguistic and indexical speech perception skills in school-age children with early cochlear implantation. Ear & Hearing, 2013 Sep; 34(5): 562-574.

28.Govaerts PJ, De Beukelaer C, Daemers K, De Ceulaer G, Yperman M, Somers T, Schatteman I, Offeciers FE. Outcome of cochlear implantation at different ages from 0 to 6 years. Otol Neurotol. 2002 Nov;23(6):885-90.

29.Hamza Y, Okalidou A, Dierckx A, van Wieringen A. Sonority-Related Novel Word Learning Ability of Children With Cochlear Implants With Optimal Oral Language Exposure. Ear Hear. 2020 Nov/Dec;41(6):1715-1731.

30.Hashemi, S.B., Monshizadeh, L. Expressive language development in 45 cochlear implanted children following 2 years of implantation (2011). Iranian Rehabilitation Journal, 9 (14), pp. 14-17.

31.Hayes H, Geers AE, Treiman R, Moog JS. Receptive vocabulary development in deaf children with cochlear implants: achievement in an intensive auditory-oral educational setting. Ear Hear. 2009 Feb;30(1):128-135.

32.Hess, Christi; Zettler-Greeley, Cynthia; Godar, Shelly P; Ellis-Weismer, Susan; Litovsky, Ruth Y. The effect of differential listening experience on the development of expressive and receptive language in children with bilateral cochlear implants. Ear & Hearing, Jul/Aug2014; 35(4): 387-395.

33. Higgins MB; McCleary EA; Carney AE; Schulte L Longitudinal changes in children's speech and voice physiology after cochlear implantation. Ear & Hearing, Feb2003; 24(1): 48-70.

34. Holt R., Beer J., Kronenberger, W., Pisoni, D., Lalonde,K., Mulinaro, L. Family Environment in Children with Hearing Aids and Cochlear Implants: Associations with Spoken Language, Psychosocial Functioning, and Cognitive Development. Ear Hear. 2020 ; 41(4): 762–774.

35. Holt, Rachael Frush; Beer, Jessica; Kronenberger, William G; Pisoni, David B. Developmental effects of family environment on outcomes in pediatric cochlear implant recipients. Otology & Neurotology, 2013 Apr; 34(3): 388-395.

36.Horn DL; Fagan MK; Dillon CM; Pisoni DB; Miyamoto RT; Horn, David L; Fagan, Mary K; Dillon, Caitlin M; Pisoni, David B; Miyamoto, Richard T. Visual-motor integration skills of prelingually deaf children: implications for pediatric cochlear implantation. Laryngoscope, 2007; 117(11): 2017-2025.

37.Hyde M, Punch R. The modes of communication used by children with cochlear implants and the role of sign in their lives. Am Ann Deaf. 2011 Winter;155(5):535-549.

38.Hyde M, Punch R, Komesaroff L. A comparison of the anticipated benefits and received outcomes of pediatric cochlear implantation: parental perspectives. Am Ann Deaf. 2010 Summer;155(3):322-338.

39.Johnson, Carol; Goswami, Usha Phonological Awareness, Vocabulary, and Reading in Deaf Children With Cochlear Implants. Journal of Speech, Language and Hearing Research (Online); Rockville Vol. 53, Iss. 2. (Apr 1, 2010): 237-261.

40.Koester, Anjali Carrasco; Mailloux, Zoe; Coleman, Gina Geppert; Mori, Annie Baltazar; Paul, Steven M; et al. Sensory Integration Functions of Children With Cochlear Implants, The American Journal of Occupational Therapy; Bethesda Vol. 68, Iss. 5 (Sep/Oct 2014): 562-569.

41.Leigh, Jaime; Dettman, Shani; Dowell, Richard; Sarant, Julia. Evidence-based approach for making cochlear implant recommendations for infants with residual hearing. Ear & Hearing, 2011 May-Jun; 32(3): 313-322.

42.Liu H, Jin X, Li J, Liu L, Zhou Y, Zhang J, Ge W, Ni X. Early auditory preverbal skills development in Mandarin speaking children with cochlear implants. Int J Pediatr Otorhinolaryngol. 2015 Jan;79(1):71-75.

43.Lonka E, Hasan M, Komulainen E. Spoken language skills and educational placement in Finnish children with cochlear implants. Folia Phoniatr Logop. 2011;63(6):296-304.

44.Marnane V, Ching TY. Hearing aid and cochlear implant use in children with hearing loss at three years of age: Predictors of use and predictors of changes in use. Int J Audiol. 2015 Aug;54(8):544-51.

45. May-Mederake B. Early intervention and assessment of speech and language development in young children with cochlear implants. Int J Pediatr Otorhinolaryngol. 2012 Jul;76(7):939-46.

46.Moberly, Aaron C.; Lowenstein, Joanna H.; Nittrouer, Susan. Early Bimodal Stimulation Benefits Language Acquisition for Children With Cochlear Implants. Otology & Neurotology, Jan2016; 37(1): 24-30.

47: Percy-Smith L, Tønning TL, Josvassen JL, Mikkelsen JH, Nissen L, Dieleman E, Hallstrøm M, Cayé-Thomasen P. Auditory verbal habilitation is associated with improved outcome for children with cochlear implant. Cochlear Implants Int. 2018 Jan;19(1):38-45.

48.Percy-Smith, L., Hall-strom, M., Josvassen, J., Mikkelsen, J., Nissen, L., Dieleman, E., & Caye-Thomasen, P. 2018. Differences and similarities in early vocabulary development between children with hearing aids and children with cochlear implant enrolled in 3-year auditory verbal intervention. International Journal of Pediatric Otorhinolaryngology, 108, 67-72.

49.Quittner AL; Cruz I; Barker DH; Tobey E; Eisenberg LS; Niparko JK; Quittner, Alexandra L; Cruz, Ivette; Barker, David H; Tobey, Emily; Eisenberg, Laurie S; Niparko, John K. Effects of maternal sensitivity and cognitive and linguistic stimulation on cochlear implant users' language development over four years. Journal of Pediatrics, Feb2013; 162(2): 343-348.

50. Sahli AS. Developments of children with hearing loss according to the age of diagnosis, amplification, and training in the early childhood period. Eur Arch Otorhinolaryngol. 2019 Sep;276(9):2457-2463.

51.Sharma, S., Bhatia, K., Singh, S., Lahiri, A., Aggarwal, A. (2017). Impact of socioeconomic factors on paediatric cochlear implant outcomes. International Journal of Pediatric Otorhinolaryngology, 102-90-97.

52.Sininger, Yvonne S.; Grimes, Alison; Christensen, Elizabeth. Auditory development in early amplified children: factors influencing auditory-based communication outcomes in children with hearing loss. Ear & Hearing, 2010 Apr; 31(2): 166-185.

53.Sparreboom, Marloes; Snik, Ad F M; Mylanus, Emmanuel A Sequential Bilateral Cochlear Implantation in Children: Development of the Primary Auditory Abilities of Bilateral Stimulation, M. Audiology & Neurotology; Basel Vol. 16, Iss. 4, (May 2011): 203-13.

54.Suskind, Dana L.; Graf, Eileen; Leffel, Kristin R.; Hernandez, Marc W.; Suskind, Elizabeth; Webber, Robert; Tannenbaum, Sally; Nevins, Mary Ellen. Project ASPIRE: Spoken Language Intervention Curriculum for Parents of Low-socioeconomic Status and Their Deaf and Hard-of-Hearing Children. Otology & Neurotology, Feb2016; 37(2): e110-e117.

55.Szagun G, Stumper B. Age or experience? The influence of age at implantation and social and linguistic environment on language development in children with cochlear implants. J Speech Lang Hear Res. 2012 Dec;55(6):1640-54.

56.Spencer LJ, Guo LY. Consonant development in pediatric cochlear implant users who were implanted before 30 months of age. J Deaf Stud Deaf Educ. 2013 Jan;18(1):93-109.

57.Tajudeen BA; Waltzman SB; Jethanamest D; Svirsky MA; Tajudeen, Bobby A; Waltzman, Susan B; Jethanamest, Daniel; Svirsky, Mario A. Speech perception in congenitally deaf children receiving cochlear implants in the first year of life.Otology & Neurotology, 2010 Oct; 31(8): 1254-1260.

58.Thomas, E. & Zwolan, T. (2019). Communication mode and speech and language outcomes of young cochlear implant recipients: A comparison of auditory-verbal, oral communication, and total communication. Otology & Neurotology, 40, 1-9.

59.Tomblin, J Bruce; Barker, Brittan A; Spencer, Linda J; Zhang, Xuyang; Gantz, Bruce J. The Effect of Age at Cochlear Implant Initial Stimulation on Expressive Language Growth in Infants and Toddlers, Journal of Speech, Language, and Hearing Research; Rockville Vol. 48, Iss. 4, (Aug 2005): 853-67.

60.Tye-Murray N. Conversational fluency of children who use cochlear implants. Ear & Hearing, 2003 Feb Suppl; 24(1): 82S-9S.

61.Verhoeven J, Hide O, De Maeyer S, Gillis S, Gillis S. Hearing impairment and vowel production. A comparison between normally hearing, hearing-aided and cochlear implanted Dutch children. J Commun Disord. 2016 Jan-Feb;59:24-39.

62.Von Muenster, K. & Baker, et al. (2014). Oral communicating children using a cochlear implant: Good reading outcomes are linked to better language and phonological processing abilities. International Journal of Pediatric Otorhinolaryngology, 78(3), 433-444.

63.Wang, Nan-Mai; Liu, Chu-Jung; Liu, Shu-Yu; Huang, Kuo-You; Kuo, Yu-Ching. Predicted factors related to auditory performance of school-aged children with cochlear implants. Cochlear Implants International: An Interdisciplinary Journal, May2011 Supplement; 12(S1): S92-5.

64.Wie OB, Falkenberg ES, Tvete O, Tomblin B. Children with a cochlear implant: characteristics and determinants of speech recognition, speech-recognition growth rate, and speech production. Int J Audiol. 2007 May;46(5):232-43.

65.Wiefferink, K., De Raeve, L., Spaai, G., Vermeij, B., Uilenburg, N. (2008). Influence of Linguistic Environment on Children's Language Development: Flemish versus Dutch Children. Deafness and Education International, 10 (4), 226-243.

66.Yanbay, E., Hickson, L., Scarinici, N., Constantinescu, G., & Dettman, S. (2014). Language outcomes for children with cochlear implants enrolled in different communication programs. Cochlear Implants International, 15 (3), 121-135.
