# Supplementary material for: Quality Standard for Rehabilitation of Young Deaf Children Receiving Cochlear Implants
Source: Medicina (Kaunas). 2023 Jul 24;59(7):1354. doi: 10.3390/medicina59071354 (PMC10386159; doi:10.3390/medicina59071354)
Supplement: Supplementary file 1 [file medicina-59-01354-s001.zip › Table S1 and Table S2.pdf]

medicina

MDPI

**Table S1.** Content of the standardized interview.

| | **Questions address to local experts** |
|---|---|
| 1 | What is your experience in rehabilitation of young deaf children with CI's learning to speak and to develop spoken language? |
| 2 | Who (what kind of professionals/parents) should be involved in the rehabilitation process after CI in young deaf children? |
| 3 | What should be the role for the CI-team and what should we expect from local professionals? So, who is doing what? |
| 4 | What are important domains to focus on during the rehabilitation of young CI-children? |
| 5 | Should we involve the parents in the rehabilitation process? If so, Why + How + How often? |
| 6 | Do you prefer a specific rehabilitation program or approach for therapy? Which resources (books, publications) do you suggest for other therapists to become an expert too? |
| 7 | Do we have to monitor the listening, speech and spoken language development of these young CI-children? If so. Why and how often? |
| 8 | What frequency of therapy sessions do you suggest for these young CI-children and their families? How often? For how many minutes? For how many months/years? |

**Table S2.** Quality standard for rehabilitation of young children receiving CI's.

| **General quality standards for pediatrics cochlear implantation in relation to rehabilitation** | **Quality standards on fitting/programming in relation to rehabilitation** | **Quality standards on rehabilitation of young children receiving CI's** | **Quality standards for professionals in relation to rehabilitation** |
|---|---|---|---|
| 1. Providing a child with a cochlear implant requires a dedicated multi-disciplinary team consisting of at least an otolaryngologist, audiologist and a speech and language specialist. | 1. The fitting of the sound processor should be carried out by qualified pediatric audiologist preferably in clinic, face-to-face rather than remotely. | 1. Rehabilitation should begin before implantation and at the latest immediately after initial fitting, according to the individual needs of the child. | 1. Every country should have training opportunities for professionals in the various communication approaches (from auditory verbal to sign bilingualism) to become an expert in the field of rehabilitation and education of CI-children. |
| 2. The CI-team of the hospital should coordinate the selection, surgery, fitting, rehabilitation and after care (equipment maintenance, spare materials). | 2. There should be a liaison between the audiologist of the CI-team and the local rehabilitation expert/local support team (and vice versa) to exchange information about the progress of the child's auditory skills. | 2. Parents/educators/professionals are considered and valued as equal partners in the rehabilitation process of their child. | 2. The staff of the CI team in the hospital and local rehabilitation therapists should have the knowledge and expertise that enables them to work effectively with children wearing CI's, including those with additional needs than their hearing loss. |
| 3. Parents/educators/professionals need balanced and unbiased up-to-date information about | 3. Instructions on the use of the sound processor must be given to the parent/caregiver on or before the day of activation and should | 3. Appropriate measures should be performed yearly (ideally every 6 months) to monitor progress in language, communicational and | 3. Rehabilitation of young CI-children should be carried out by an expert in promoting listening, speech and spoken |

| | | | |
|---|---|---|---|
| CI's and the fitting/rehabilitation process. | be repeated at least twice within the six months following activation. | educational outcomes the first 3 years after implantation. | language development, in managing the technology and the environment. |
| 4. Rehabilitation should be delivered by the CI-team in close cooperation with a local expert (team) in listening and spoken language development. (see quality standard # 29) | 4. Appropriate audiological, standardized speech perception tests and functional hearing assessment (by family/other professionals' questionnaire) should be performed at 6 months intervals to enable hearing to be monitored. | 4. A diagnostic coaching approach to CI rehabilitation yields the most efficient and best benefit, both to children and to parents/ educators/professionals. | 4. The expertise of the rehabilitation therapist/rehabilitation team should include certain skills. |
| 5. Rehabilitation is not possible without parent/family/caregiver involvement | | 5. The audiologist and speech and language therapist together with the parents should decide on the frequency of specialist contact sessions for fitting and for rehabilitation based on the individual needs of the child and their family. | |
| 6. The cochlear implant surgery should take place as soon as a child is identified as a candidate and should ideally be done by the age of 12 months or sooner, preferably under the age of 36 months, without excluding children who are older than 37 months. | | 6. As the recommended approach of services is family-centered, it is understood that rehabilitation therapy sessions can take place weekly or fortnightly, considering that most listening and spoken language experience will occur at home between the sessions. | |
| 7. A child with a bilateral severe to profound hearing loss should be fitted bilaterally with CI's, preferably before the age of 18 months. | | 7. Children with CI's should have annually the opportunity to trial and assess assistive listening devices (FM-systems, Bluetooth accessories). | |
| 8. The CI-team will issue or dispatch replacements for faulty external equipment within two working days. | | 8. Rehabilitation of young CI-children should involve collaboration between the CI Centre, local professionals, and parents/educators to cover specific areas. | |
| 9. Arrangements should be in place to upgrade each child's sound processor every 5 years. | | 9. Although services differences, based on each child's current level of performance, it is recommended that children receive listening and spoken language therapy after implantation to maximize | |

| | | | |
|---|---|---|---|
| | | benefit from the CI's, even those who benefit little from CI and who are anticipated to still be sign reliant. | |
| 10. The implant program should perform and publish yearly audits and comply with the requirements of the responsible national authorities. Audits should cover: clinical activity, staffing levels of expertise, child's performance outcomes, medical and surgical complications, device failures, research outcomes and child and family/caregiver feedback on the service provided. | | 10. Rehabilitation therapists and parents/ educators will collaboratively generate measurable and appropriate goals in all areas of the child's development (auditory, receptive, and expressive language, speech, cognition, and social skills), and identify ways to integrate the goals and strategies to achieve them in a nurturing and rich language. | |
| | | 11. Music should be integrated in the rehabilitation of young children using CI's, particularly as a home-based fun activity rather than in a formal setting. | |
